# *Mycoplasma pneumonia* Infection Is Associated With an Increased Risk of Systemic Lupus Erythematosus: A Nationwide, Retrospective Cohort Study

**DOI:** 10.3389/fmicb.2022.815136

**Published:** 2022-04-21

**Authors:** Kuo-An Chu, Ting-Yun Ou, Wei-Hsin Hung, Jie Sung, Weishan Chen, Cheng-Li Lin, Yao-Min Hung, James Cheng-Chung Wei

**Affiliations:** ^1^Division of Chest Medicine, Department of Internal Medicine, Kaohsiung Veterans General Hospital, Kaohsiung, Taiwan; ^2^Department of Nursing, Shu-Zen Junior College of Medicine and Management, Kaohsiung, Taiwan; ^3^Institute of Biopharmaceutical Sciences, National Sun Yat-sen University, Kaohsiung, Taiwan; ^4^Department of Internal Medicine, Kaohsiung Municipal United Hospital, Kaohsiung, Taiwan; ^5^Department of Obstetrics and Gynecology, Kaohsiung Veterans General Hospital, Kaohsiung, Taiwan; ^6^Management Office for Health Data, China Medical University Hospital, Taichung, Taiwan; ^7^College of Health and Nursing, Meiho University, Pingtung, Taiwan; ^8^School of Medicine, National Yang Ming University, Taipei, Taiwan; ^9^Institute of Medicine, Chung Shan Medical University, Taichung, Taiwan; ^10^Division of Allergy, Immunology and Rheumatology, Chung Shan Medical University Hospital, Taichung, Taiwan; ^11^Graduate Institute of Integrated Medicine, China Medical University, Taichung, Taiwan

**Keywords:** *Mycoplasma pneumonia* infection, systemic lupus erythematosus, risk factor, cohort study, autoimmune disease

## Abstract

**Background:**

Infections may play a role in the development of systemic lupus erythematosus (SLE).

**Objective:**

To assess the link between *Mycoplasma pneumonia* (*M. pneumonia*) infection and the incidence of SLE.

**Method:**

We conducted a retrospective cohort study, which identified 116,043 hospitalized patients with *M. pneumoniae* between 2000 and 2012 from the Taiwan National Health Insurance Research Database and compared them with 447,839 matched inpatients who had never been diagnosed with *M. pneumonia* infection (at a 1:4 ratio, matched by age, gender, and index year). Their comparative risk of developing SLE was evaluated. The follow-up period was defined as the time from the initial diagnosis of *M. pneumonia* infection to the date of SLE diagnosis, or December 31, 2013. The incidence rates of SLE were assessed in people with and without *M. pneumoniae* infection. Cox proportional hazard models were used to estimate the hazard ratios (HRs) and 95% confidence intervals (CIs), with the uninfected group used as the reference.

**Results:**

The adjusted HR of SLE for the *M. pneumoniae* group was 2.97 with 95% CI = 2.18–4.05 compared with the uninfected group. The risk was most significantly higher within 0.5 years after the *M. pneumoniae* infection with an adjusted HR of 6.18 (95% CI = 3.82–9.97, *p* < 0.01). The adjusted HR for SLE from 0.5 to 2 years and from 2 to 5 years after *M. pneumoniae* infection was 1.59 (95% CI = 0.70–3.59, *p* = 0.27) and 2.42 (95% CI = 1.22–4.81, *p* = 0.01), respectively.

**Conclusion:**

The incidence of SLE was significantly higher in subjects infected with *M. pneumoniae*.

## HIGHLIGHTS

- Previous studies reported that individuals infected with *Mycoplasma* species were 16 times more likely to contract systemic lupus erythematosus (SLE) than control groups (Wang et al., 2011).
- Patients with a history of *Mycoplasma pneumoniae* infection are associated with a 2.97-fold increased risk of SLE. Among the different age groups, patients older than 65 years were shown to be at greatest risk.
- A significant risk (2.42-fold increase) of developing SLE was observed between 2 and 5 years after a diagnosis of *M. pneumoniae* infection compared with the uninfected group.

## Introduction

Systemic lupus erythematosus (SLE) is an autoimmune disease that affects multiple organ systems. It is more common in women, with a peak age range from the third to seventh decades of life (Rees et al., 2017). The incidence of SLE in adults ranges from 0.3 to 23.7 cases per 100,000 person-years around the world (Fessel, 1988). In Taiwan, the annual incidence of SLE was estimated to be 4.87 per 100,000 person-years in 2003–2008 and 0.74–1 per 100,000 person-years in 2001–2011 (Yeh et al., 2013; Leong et al., 2021). The etiology of SLE is still unclear, but infections can become environmental primers, inducing or promoting the onset and exacerbations of SLE in genetically susceptible individuals (Esposito et al., 2014; Rigante et al., 2014; Jung and Suh, 2017). The pathogenesis of infections is known to cause immune responses in genetically susceptible individuals through molecular simulation, epitope spreading, bystander activation, and other mechanisms (Esposito et al., 2014). Extensive research on experimental animal models clearly shows that infectious agents can destroy immunological tolerance to self-antigens and induce autoimmune diseases, mainly SLE (Rigante and Esposito, 2015). However, there has been a significant lack of epidemiological studies into bacterial infections associated with SLE.

*Mycoplasma pneumoniae* is one of the most common causes of atypical pneumonia in the United States and other parts of the world (Taylor-Robinson, 1996; Atkinson et al., 2008). *M. pneumoniae* bacteria lack a cell wall and are the smallest free-living organisms that can live alone in nature. More than 15 *Mycoplasma* species have been isolated from humans, and only four of them have been identified as human pathogens (Taylor-Robinson, 1996; Atkinson et al., 2008). *M. pneumoniae* can penetrate the cell membrane of host cells and invade the respiratory mucosa, leading to inflammatory responses and also spreading outside the respiratory system. By direct and indirect immune-mediated mechanisms, *M. pneumoniae* infection can cause *M. pneumoniae*–related extrapulmonary diseases. An indirect mechanism to the urinary tract includes low C3-complement immune-mediated injury by deposition of immune complexes. Nephritis caused by this mechanism may be one manifestation of SLE (Poddighe, 2018). Although most cases of pneumonia caused by *M. pneumoniae* are mild and self-limiting, fulminant cases have occurred. Antibodies against *Mycoplasma* may cross react with human antigens, and this is thought to promote the production of clinical symptoms from autoantibodies. Rheumatologic symptoms, such as polyarthralgia and myalgias, are thought to be caused by immune-mediated mechanisms (Chaudhry et al., 2003). The main etiology of infections as a trigger factor for SLE is pathogen-induced abnormal immune responses, such as upregulation of the type I interferon (IFN) system and superantigen production, leading to activation of T lymphocytes (Barbhaiya and Costenbader, 2016; Pan et al., 2019). Many studies have found that Epstein–Barr virus (EBV) infections are more common in patients with SLE, and the seropositive rates of EBV are much higher in these patients (Jung and Suh, 2017). Although the association between cytomegalovirus (CMV) infection and SLE remains controversial, CMV antibodies were higher in patients with SLE compared with normal controls in recent studies (Barbhaiya and Costenbader, 2016). In addition to viral infection, bacterial infection, such as vibrio cholera and group A *Streptococcus* (GAS), may also trigger SLE; however, its pathogenesis is less well-understood (Pan et al., 2019).

In Taiwan, the main diagnostic criterion for *M. pneumoniae* infection is a seropositive finding for *M. pneumoniae* immunoglobulin M (IgM) or a fourfold increase in anti–*M. pneumoniae* immunoglobulin G (IgG) (Lee et al., 2017). Use of multiplex real-time polymerase chain reaction for the detection of *M. pneumoniae* has been increasing recently. There are many risk factors that may cause *M. pneumoniae* infection, including old age, younger age in children less than 2 years old, tobacco smoking, chronic lung disease, malignancies, and immunocompromised disease, such as human immunodeficiency virus (HIV) infection (Reynolds et al., 2010).

Another prospective study was conducted in 168 adults, in 13 Taiwanese hospitals in 2001–2002, which revealed that the most common pathogen for community-acquired pneumonia was *Streptococcus pneumoniae*, followed by *M. pneumoniae* (Lauderdale et al., 2005). Considering that *M. pneumoniae* is the second most common cause of community-acquired bacterial pneumonia in Taiwan (Peto et al., 2014) and because pneumonia is among the most common infections and the third leading cause of mortality in Taiwan, we conducted a study aimed at assessing the link between *M. pneumoniae* infection and SLE incidence.

## Materials and Methods

### Study Design

This study was a retrospective cohort study.

### Data Source and Study Period

The data used in this study was from the Taiwan National Health Insurance Research (NHIRD) database and was released by the National Research Institute for research purposes. The Taiwan NHI program was established in 1995 and currently covers greater than 99% of Taiwan’s population. Data in the NHIRD include hospitalization expenses calculated by admission and the hospitalization records of all beneficiaries participating in the NHI program. We also used the catastrophic illness database to ensure that the diagnoses of SLE were reliable. To ensure the privacy of patients, their identities were encrypted before being released by the National Research Institute. This study was approved by the Institutional Review Board (IRB) and the Hospital Research Ethics Committee at China Medical University (IRB permit no. CMUH-104-REC2-115; valid date of research project: from July 22, 2021 to July 21, 2022).

The main outcome was diagnosis of SLE, which was classified as a catastrophic illness in Taiwan. SLE was diagnosed by experienced rheumatologists by carefully reviewing the original medical records, laboratory data, imaging manifestations, and pathological results of all patients who applied for a catastrophic illness certificate in Taiwan. Rheumatologists in Taiwan followed the 1982 American College of Rheumatology classification criteria and the 1997 revision criteria for SLE diagnosis between 2000 and 2013 (Aringer et al., 2020). The Bureau of NHI issues certificates of catastrophic illness only to those who meet the SLE criteria: *International Classification of Diseases, Ninth Revision, Clinical Modification* (Cheng et al., 2011) (*ICD-9-CM*) code 710.0. The patients were followed from 2000 until a diagnosis of SLE, withdrawal from the NHI program, or the end of 2013, whichever occurred first.

### Study Population

The subjects included within this study were hospitalized patients infected or uninfected with *M. pneumoniae* (*ICD-9-CM* code 483.0), from 2000 to 2013. The index date was defined as the admission date with the diagnosis of *M. pneumoniae* infection. Patients previously diagnosed with SLE were excluded. Those who withdrew from the NHI program before the index date were also excluded. The exposed group consisted of hospitalized patients with a diagnosis of *M. pneumoniae* infection, and the uninfected group consisted of those who did not have that infection. A total of 116,043 patients were included in the *M. pneumoniae* infection group, and 447,839 patients were included in the uninfected group.

### Data Collection

The data were retrospectively collected from hospitalized patients in the Taiwan NHI database from 2000 to 2013. We collected data on *M. pneumoniae* infection, SLE diagnosis, and comorbidities.

### Statistical Analysis

The incidence rate was calculated based on the number of occurrences and person-years. Person-years were calculated as the sum of the follow-up time for each individual, and the follow-up time was defined as the period from the index date to the diagnosis of SLE, withdrawal from the NHI program, or the end of 2013. Univariate and multivariate Cox proportional hazard regression models were used to estimate hazard ratios (HRs) and 95% confidence intervals (CIs) in the two groups. The variables in the multivariate model included age, gender, and all comorbidities. The Kaplan–Meier method was used to describe the cumulative incidence of SLE in the two groups; the log-rank test was used to assess differences between the two groups. The χ^2^ test was used for categorical variables, and the *t*-test was used for continuous variables. SAS statistical software (version 9.4 for Windows; SAS Institute Inc., Cary, NC, United States) was used for the data analysis, and *p* < 0.05 was considered to indicate a statistically significant difference.

## Results

### Baseline Characteristics of the Study Population

The eligible study participants included 116,043 patients in the *M. pneumoniae* infection group and 447,839 patients in the uninfected group. There was a significant difference in age and gender between the two groups due to the large sample size and statistical power (Table 1). There were more females (52.0%) than males, and most patients were ≤19 years old (85.5%). The *M. pneumoniae* infection group had a higher incidence rate of comorbidities (Table 2), which was compatible with the risk factors for *M. pneumoniae* infection. The mean follow-up time in the *M. pneumoniae* infection group and the uninfected group was 5.19 and 5.21 years, respectively.

**TABLE 1:** Baseline characteristics of the study population.

	*Mycoplasma pneumonia* infection				*p*-value*
	Yes		No		
	(*n* = 116,043)		(*n* = 447,839)		
	n	%	n	%	
**Gender**					<0.01
Male	55,747	48.0	219,744	49.1	
Female	60,296	52.0	228,095	50.9	
**Age (y)**					0.00
≦19	99,240	85.5	380,630	85.0	
20–39	7,026	6.05	28,104	6.28	
40–64	4,918	4.24	19,672	4.39	
≧65	4,859	4.19	19,433	4.34	
Mean (SD)	12.0 (18.0)		11.6 (18.6)		<0.0001^a^
**Comorbidity**					
Hypertension	4,827	4.16	11,085	2.48	<0.01
Diabetes mellitus	2,824	2.43	6,533	1.46	<0.01
Hyperlipidemia	1,430	1.23	2,114	0.47	<0.01
CAD	2,307	1.99	2,747	0.61	<0.01
Hepatitis B	623	0.54	1,031	0.23	<0.01
Hepatitis C	479	0.41	494	0.11	<0.01
Cancer	855	0.74	1,482	0.33	<0.01
Allergic rhinitis	7,429	6.40	6,904	1.54	<0.01
Chronic liver diseases	437	0.38	160	0.04	<0.01
Atopic dermatitis	7,468	6.44	9,627	2.15	<0.01
Asthma	19,385	16.7	10,539	2.35	<0.01
COPD	3,000	2.59	1,294	0.29	<0.01

*CAD, coronary artery disease; COPD, chronic obstructive pulmonary disease.*

*χ^2^ Test.

*^a^t-test.*

**TABLE 2:** Incidence and hazard ratio of systemic lupus erythematosus in the study cohort.

	*Mycoplasma pneumonia* infection						Compared with uninfected group			
	Yes			No			Crude		Adjusted	
	Occurrence of SLE	PY	IR	Occurrence of SLE	PY	IR	HR (95% CI)	*p*-value	HR (95% CI)	*p*-value
Overall	67	602,130	11.1	110	2,333,202	4.7	2.37 (1.75–3.21)	0.62	2.97 (2.18–4.05)	<0.01
**Gender**										
Male	9	287,696	3.13	15	1,141,087	1.31	2.37 (1.04–5.42)	0.04	3.5 (1.46–8.38)	<0.01
Female	58	314,434	18.4	95	1,192,115	8.0	2.33 (1.68–3.22)	<0.01	2.33 (1.68–3.22)	<0.01
**Age (y)**										
≦19	32	535,586	5.97	87	2,055,124	4.23	1.42 (0.94–2.12)	0.09	1.69 (1.12–2.55)	0.01
20–39	23	33,724	68.2	12	133,819	9.0	7.65 (3.81–15.4)	<0.01	8.90 (4.42–17.9)	<0.01
40–64	8	19,886	40.2	7	82,672	8.47	4.68 (1.70–12.9)	<0.01	5.63 (1.95–16.2)	<0.01
*geqq*65	4	12,935	30.9	4	61,587	6.5	4.31 (1.08–17.3)	0.04	9.62 (2.16–42.9)	<0.01
Comorbidity*										
No	56	428,395	13.1	103	2,126,944	4.8	2.72 (1.96–3.76)	<0.01	2.80 (2.02–3.89)	<0.01
Yes	11	173,734	6.33	7	206,258	3.39	1.91 (0.74–4.92)	0.18	4.90 (1.75–13.7)	<0.01
Hypertension	4	14,028	28.5	6	38,276	15.6	1.72 (0.49, 6.09)	0.40	2.78 (0.75, 10.3)	0.13
Diabetes mellitus	0	8,270	0	2	23,861	8.38	—	—	—	—
Hyperlipidemia	1	4,425	22.6	0	8,429	0.00	—	—	—	—
CAD	0	6,635	0.00	2	10,000	20.0	—	—	—	—
Hepatitis B	1	2,315	43.2	0	3,950	0.00	—	—	—	—
Hepatitis C	1	1,350	74.1	0	1,803	0.00	—	—	—	—
Cancer	0	2,154	0	0	4,448	0	—	—	—	—
Allergic rhinitis	1	36,035	2.78	1	34,840	2.87	0.97 (0.06, 15.5)	0.98	2.83 (0.09, 87.5)	0.55
Chronic liver diseases	0	1,645	0.00	0	829	0.00	—	—	—	—
Atopic dermatitis	1	38,131	2.62	0	50,421	0.00	—	—	—	—
Asthma	2	104,004	1.92	0	57,533	0.00	—	—	—	—
COPD	1	8,865	11.3	0	3,897	0.00	—	—	—	—

*CI, confidence interval; HR, hazard ratio; IR, incidence rate, per 100,000-person years; PY, person-years.*

*Model was adjusted by gender, age, and all comorbidities listed in Table 1.*

*Patients with any one of the comorbidities were classified as the comorbidity group.

### Systemic Lupus Erythematosus Incidence

The overall incidence rates of SLE in the *M. pneumoniae* infection and uninfected groups were 11.1 and 4.7 per 100,000-person years, respectively. After stratifying by gender, the female incidence rates of SLE were 18.4 and 8.0 per 100,000 person-years in the infected and uninfected groups, respectively. In males, the incidence rates of SLE were 3.13 and 1.31 per 100,000 person-years, respectively. After stratifying by age, the incidence rate of SLE was highest in the 20–39-year age group: 68.2 per 100,000 person-years vs. 9 per 100,000 person-years in the infected and uninfected groups, respectively.

### Systemic Lupus Erythematosus Risk Due to *Mycoplasma pneumoniae* Infection

**FIGURE 1:**
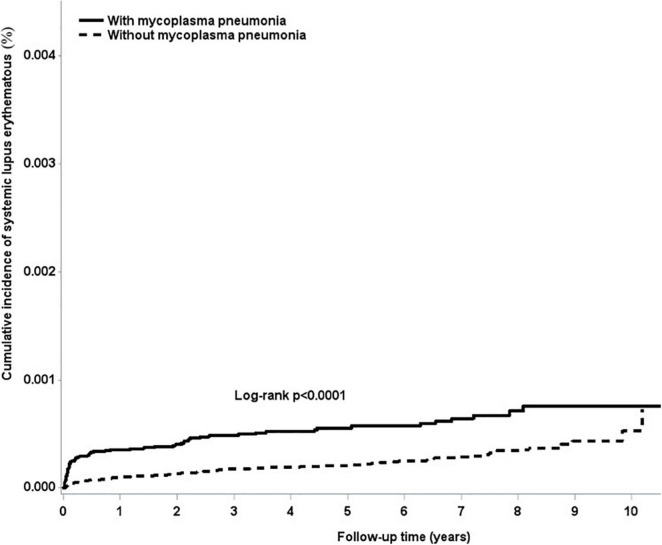
Cumulative incidence of systemic lupus erythematosus in the study cohort.

The overall adjusted HR for developing SLE was 2.97 (95% CI = 2.18–4.05) in subjects who were infected with *M. pneumoniae* compared with the uninfected subjects. After stratifying by gender, females infected with *M. pneumoniae* had a 2.33-fold (95% CI = 1.68–3.22) higher risk of developing SLE, whereas males had a 3.5-fold (95% CI = 1.46–8.38) higher risk of developing SLE, compared with their non-infected counterparts (Table 3). Figure 1 shows that the cumulative incidence of SLE was significantly higher in the *M. pneumoniae* group compared with the uninfected group (*p* < 0.01). An overlap of SLE cumulative incidence between the *M. pneumoniae* group and the uninfected group may be due to the small sample size. After stratifying by age, the risk of SLE following *M. pneumoniae* infection was highest in the >65- and 20- to 39-year age groups: HR = 9.62 (95% CI = 2.16–42.9) and 8.90 (95% CI = 4.42–17.9), respectively. After stratifying by comorbidity status, patients with comorbidities had a 4.9-fold (95% CI = 1.75–13.7) higher risk of developing SLE following *M. pneumoniae* infection compared with those without comorbidities. After stratifying by study period, the risk of SLE was significantly higher in the <0.5 and 2–5 years following *M. pneumonia*e infection: HR = 6.18 (95% CI = 3.82–9.97) and 2.42 (95% CI = 1.22–4.81), respectively (Table 4).

**TABLE 3:** Incidence and hazard ratio of systemic lupus erythematosus according to the duration of follow-up.

	*Mycoplasma pneumonia* infection								Compared to uninfected group			
	Yes				No				Crude		Adjusted	
	n	Occurrence of SLE	PY	IR	n	Occurrence of SLE	PY	IR	HR (95% CI)	*p*-value	HR (95% CI)	*p*-value
**Follow time**												
<0.5	116,043	38	57,648	65.9	447,839	32	222,454	14.4	4.58 (2.86–7.33)	<0.01	6.18 (3.82–9.97)	<0.01
0.5-2	114,814	8	165,464	4.83	443,558	25	637,104	3.92	1.23 (0.56–2.73)	0.61	1.59 (0.70–3.59)	0.27
2-5	100,391	13	274,020	4.74	389,430	25	1,059,611	2.36	2.01 (1.03–3.94)	0.04	2.42 (1.22–4.81)	0.01
≧5	55,309	8	146,244	5.47	216,902	28	570,500	4.91	1.14 (0.52–2.50)	0.75	1.34 (0.61–2.94)	0.47

*CI, confidence interval; HR, hazard ratio; IR, incidence rate, per 100,000-person years; PY, person-years.*

*Model was adjusted by gender, age, and all comorbidities listed in Table 1.*

**TABLE 4:** The mechanism of *M. pneumoniae* infection increased SLE risk.

Mechanism of *M. pneumoniae* infection increased SLE risk	References
*Mycoplasma* arthritic mitogen (MAM) activates T-cell and induces proinflammatory cytokines	Rink et al., 1996; Shibata et al., 1997; Mu et al., 2000
*M. pneumoniae* infection produced immunoglobulin E (IgE) against polypeptide of P1 protein	Sanjuan et al., 2016; Lamri and Charles, 2020
Autoreactive IgE trigger basophils, modulating immune responses	Charles et al., 2010; Sanjuan et al., 2016; Pan et al., 2017; Poddighe and Marseglia, 2017; Lamri and Charles, 2020
Plasmacytoid dendritic cells activation, inducing IFN-α	Herrada et al., 2019
Infection induce IFN-α by membrane interactions	Capobianchi et al., 1987
Mycoplasmal lipid-associated membrane proteins (LAMPs) activate immune system	Feng and Lo, 1994; da Rocha Sobrinho et al., 2011
Mimic molecules of self-antigens by mycoplasma produce antibodies to mycoplasmal antigens	Rottem, 2003

## Discussion

To the best of our knowledge, this is the first nationwide cohort study to determine that patients with *M. pneumoniae* infection exhibited a 2.97-fold greater risk of subsequently developing SLE compared with the general population. Furthermore, the effects of *M. pneumoniae* infection were found to be significant in both genders and in all age groups of patients. A significant risk (6.18-fold) of developing SLE was also observed in the 0.5-year follow-up after a diagnosis of *M. pneumoniae* infection compared with the general population. As we know, SLE may be an indolent disease at the beginning, the diagnosis of SLE in the half-year follow-up after *M. pneumoniae* infection may be due to the frequent medical visits made by the patients because of their recent infection with *M. pneumoniae*. Thus, the causation between *M. pneumoniae* infection and SLE may not exist. Incidentally diagnosed SLE in the 0.5-year follow-up may be due to previous indolent SLE and frequent medical checks after the *M. pneumoniae* infection. It makes sense that indolent SLE increases the risk of *M. pneumoniae* infection. There is still a prominent risk (2.42-fold) of developing SLE in the 2- to 5-year follow-up after a diagnosis of *M. pneumoniae* infection. Although patients with *M. pneumoniae* infection had a significantly higher rate of comorbid diseases compared with the uninfected group, *M. pneumoniae* infection remained an independent risk factor for developing SLE in terms of gender, age, and comorbidities, especially during the 2- to 5-year follow-up.

This study can provide inspiration for current practice. After a diagnosis of *M. pneumoniae* infection, appropriate information and advice should be provided to high-risk groups to promote early medical management or specialist referral. From a public health perspective, policy makers are encouraged to strengthen screening for autoimmune diseases in patients with a history of *M. pneumoniae* infection and to provide more integrated care, such as family practice, infection specialist, and rheumatology care.

There are many infectious agents involved in the pathogenesis of SLE. The strongest evidence for this correlation is viruses, such as EBV, parvovirus B19 virus, and exogenous retroviruses (e.g., human T-lymphotropic virus type 1, HIV type 1, and endogenous retroviruses) (Pan et al., 2019). Viral-induced autoimmunity by molecular mimicry, epitope spreading, bystander activation, and immortalization of infected B cells may lead to autoimmune disease (Smatti et al., 2019). Other infections that may cause SLE are CMV, transfusion-transmitted virus, GAS, and *Vibrio cholerae* (Li et al., 2019; Pan et al., 2019; Smatti et al., 2019). A recent study reported a higher incidence of mycoplasmas in the urogenital tract of women with SLE, but the difference was not statistically significant (Alicja et al., 2020). Many previous studies have also claimed that patients with SLE have an increased likelihood of *Ureaplasma urealyticum* and *Mycoplasma hominis* infection, two genital mycoplasmas (Machado et al., 2001; Mendez-Martinez et al., 2017). *M. pneumoniae* infection–induced SLE was not presented in these previous studies; thus, it needs further study to confirm its correlation.

The underlying mechanism by which *M. pneumoniae* infection increases the risk of SLE is unclear. Mycoplasma antigens induce both cell-mediated and humoral immune responses. *In vitro* studies have shown that these *Mycoplasma* species can cause polyclonal activation of B cells and the production of superantigens (Cole and Griffiths, 1993). The *Mycoplasma* arthritic mitogen (MAM) can activate T cells and change cytokine expression profiles, activating and regulating the signaling pathways of nitric oxide synthase. MAM induces the synthesis of proinflammatory cytokines, such as interleukin (IL)-1, IL-6, and IL-8 (Rink et al., 1996; Shibata et al., 1997). In addition, the regulation of cytokine synthesis by MAM corresponds to the induction of arthritic changes in mice (Mu et al., 2000). Recent studies using flow cytometric analysis revealed that *Mycoplasma* species cause cytokines to react toward T helper 2 (Th2) and Th17, including IL-1, IL-6, IL-17, and also cause regulatory T cell (Treg) imbalance (Guo et al., 2016; Odeh and Simecka, 2016).

Th17 and Treg imbalance has been detected in various autoimmune diseases and has been shown to exacerbate inflammatory responses (Guo et al., 2016). One prospective study found that 9.2% of children hospitalized due to *M. pneumoniae* infection produced immunoglobulin E (IgE) against the polypeptide of P1 protein. These patients did not have a history of allergy and had more severe clinical symptoms and excessive secretion of IL-4 and IL-5 and overdifferentiation of Th0 cells into Th2 cells (Ye et al., 2018). The specificity of autoreactive IgE to double-stranded DNA (dsDNA), single-stranded DNA, nuclear ribonucleoproteins, and Smith antigen in SLE patient serum have been described and have the ability to trigger basophil and plasmacytoid dendritic cell activation. This autoreactive IgE overstimulates already dysregulated pathways in SLE and is associated with disease activity (Sanjuan et al., 2016; Lamri and Charles, 2020).

Activated basophils participate in some allergic reactions and immune responses to parasite infections. Basophils in SLE patients can promote antibody production by B cells and support IL-17–producing Th17 differentiation of T cells during *in vitro* study, thus promoting inflammation (Pan et al., 2017) and the development of lupus nephritis (Charles et al., 2010; Dema et al., 2017). Decreased numbers and increased activity of peripheral basophils by a constitutional Th2-shifted inflammatory environment in active SLE patients and murine models were found in different studies and may correlate with their disease activity and increased antibody production (Sanjuan et al., 2016; Dema et al., 2017; Pan et al., 2017; Poddighe and Marseglia, 2017; Liang et al., 2018; Poddighe et al., 2021). Plasmacytoid dendritic cell–induced type I IFN production after virus infection may be linked to SLE development (Herrada et al., 2019). Previous studies also showed increased serum levels of IFN-α in SLE patients (John J. Hooks et al., 1979; Ytterberg and Schnitzer, 1982). *M. pneumoniae* can induce IFN-α by membrane interactions (Capobianchi et al., 1987). Humoral immune responses against *Mycoplasma* species, which may trigger inflammation, are induced by *Mycoplasma* lipid-associated membrane proteins (LAMPs). One study detected serum profiles of IgG antibodies reactive against LAMPs of *M. hominis* PG21 and *Mycoplasma fermentans* PG18 in patients with rheumatoid arthritis (RA), those with SLE, and healthy individuals. It revealed that the number of LAMPs recognized by the antibodies of RA patients was higher than that of healthy individuals. The sera from patients with RA frequently recognized large amounts of LAMP. However, there were no differences detected between the sera from SLE patients and the controls (da Rocha Sobrinho et al., 2011).

*Mycoplasma* LAMPs can activate B lymphocytes and cause the appearance of antibodies against self-proteins, which can cross-react with *Mycoplasma* antigens (Feng and Lo, 1994). Mimic molecules of self-antigens by *Mycoplasma* species can produce antibodies against *Mycoplasma* antigens that react with self-antigens, causing autoimmune disease (Rottem, 2003). Despite these hypotheses, some individuals carrying antibodies against *Mycoplasma* antigens without immune responses to mycoplasmal LAMPs do not develop autoimmune disease (da Rocha Sobrinho et al., 2011). *M. pneumoniae*–related extrapulmonary diseases involving the skin and musculoskeletal, nervous, hematological, digestive, and renal systems by direct and indirect mechanisms will induce tissue damage related to immune cell recruitment and trigger inflammatory responses (Poddighe, 2018). These manifestations are like SLE with multiple organ involvement and disease heterogeneity.

As we mentioned previously, there are large amounts of LAMP in the sera of RA patients (da Rocha Sobrinho et al., 2011). *M. pneumoniae*–associated acute disseminated encephalomyelitis was reported with strongly positive anti-GM2 IgM antibodies, which lead to chronic motor neuropathy, amyotrophic lateral sclerosis–like disorder after ganglioside therapy, chronic demyelinating neuropathy with sensory ataxia, paraneoplastic polyradiculoneuritis, and Guillain–Barré syndrome (Xia and Chen, 2020). Another study reported that community-acquired *M. pneumoniae* infections may lead to the production of antiacetylcholine receptor antibodies that trigger myasthenia gravis autoimmune activation (Iwasa et al., 2018). These findings highlight the association between *M. pneumoniae* infections and autoimmune diseases.

Subgroup analysis in our study showed that all age groups with *M. pneumoniae* infection had an increased risk of SLE, and a significant 9.62-fold increase in the risk of developing SLE was observed in the eldest group. Some may consider that young people usually have stronger immune reactions to infections compared with the elderly; its mechanism of action remains unclear; however, we have hypothesized that Treg imbalance might be more prominent in the elderly. One prospective study in Toronto found that late-onset SLE where patients were diagnosed at age older than 50 years had more cardiovascular, renal, and ocular damage, which may have been due to the aging process (Aljohani et al., 2017). Although patients with late-onset SLE had the lowest incidence of major organ involvement, the highest prevalence of comorbidities and positive anti-dsDNA antibodies was found; this finding makes late-onset SLE more complicated (Medhat et al., 2020). Late-onset SLE patients often have insidious onset and lower disease activities but usually have poor outcomes, especially in men (Pons-Estel et al., 2010).

The cytokine reaction is usually higher in the first 2 years after infection, but T-cell memory can persist for longer (Jones et al., 2002; Bodhankar et al., 2010). It is reasonable to get SLE 2 to 5 years after *M. pneumoniae* infection under this theory. Consequently, clinicians should be aware of the increased risk of SLE in patients with *M. pneumoniae* infection and educate and monitor these patients. Another possible explanation for the association between *M. pneumoniae* infection and SLE is that the immunocompromised status of SLE patients may have existed before the onset of SLE-related symptoms and signs. As a result, *M. pneumoniae* infection should be taken as a potential “warning sign.”

An advantage of the current study was its use of nationwide data to assess the risk of SLE in patients with *M. pneumoniae* infection. Advantages of using the NHIRD in research have been described previously (Hsing and Ioannidis, 2015). They include large sample sizes, a lack of selection and participation bias, and long-term comprehensive follow-up data. Although prospective cohort studies are expensive, retrospective cohort studies using insurance data are a suitable and economical alternative. In addition, our subgroup analysis illustrated the interassociation of gender, different age groups, and comorbidities.

In interpreting our findings, several limitations should be considered. First, the *ICD-9-CM* codes used to diagnose *M. pneumoniae* infection and SLE were based on administrative claims data recorded by physicians and hospitals, rather than a prospective clinical setting. Although the NHI Bureau uses an audit mechanism to minimize diagnostic uncertainty and misclassification, inaccuracy may lead to misclassification. In the present study, the diagnosis of SLE was rigorously defined by the catastrophic illness database, thus allowing for improved diagnostic validity. The NHI Bureau issues certificates of catastrophic illness only to those who meet the SLE criteria. Before being given a catastrophic illness certificate, experienced rheumatologists carefully review the original medical records, laboratory data, imaging manifestations, and pathological results of the patient. Second, there were no data on body mass index, smoking, socioeconomic status, and family history in the NHIRD, all of which are potential confounding factors. Therefore, no further analysis of these variables was possible, although chronic obstructive pulmonary disease was used as a proxy variable for smoking, which has been used in several previous studies (Chang et al., 2014; Raaschou et al., 2016). There were also no data on the symptoms of *M. pneumoniae*–infected patients in our study. Third, the results of this study may be affected by medications, such as anticoagulants, antiplatelet drugs, and hormone replacement therapy but our data did not include medication records. Fourth, our data included only inpatients, which represents those who had severe *M. pneumoniae* infections. The data may not represent the whole group of those infected with *M. pneumoniae*, as we know it is a common outpatient disease. As we know severe disease will cause a stronger immune response, the association between SLE and severe *M. pneumoniae* infection could be firmer. However, our inpatient data did not include medication prescriptions, so we do not know about antibiotics such as macrolides, which may lead to autoimmunity. Fifth, we thought an overlap of SLE cumulative incidence between the *M. pneumoniae* infection group and the uninfected group may be due to the small sample size, and this observation may not completely support the fact that *M. pneumoniae* infection is critically involved in the greatest part of SLE cases. Finally, our enrollees were almost all Taiwanese. Thus, our findings may not be applicable to non-Asian ethnic groups. Because the incidence of specific SLE may differ by ethnicity and geographic location, further research should be validated in other ethnic groups. Nevertheless, our findings suggest the possible pathogenesis of SLE, which may increase the overall understanding of the diseases and lead to better patient care.

## Conclusion

This population-based cohort study demonstrated a higher risk of SLE in patients with *M. pneumoniae* infection, among both genders and those with or without any comorbidities. The risk was prominent in the first 0.5-year follow-up after a diagnosis of *M. pneumoniae* infection, which may be due to previous indolent SLE and frequent medical checks after *M. pneumoniae* infection. The adjusted HR was still high at 2.42 (95% CI = 1.22–4.81, *p* = 0.01) during the follow-up 2–5 years after *M. pneumoniae* infection. Future studies are required to clarify the underlying biological mechanisms of these associations. Clinicians should be made aware of the increased risk of SLE in patients with *M. pneumoniae* infection and should provide appropriate monitoring for high-risk groups in addition to treating the *M. pneumoniae* infection.

## Data Availability Statement

The original contributions presented in the study are included in the article/supplementary material, further inquiries can be directed to the corresponding authors.

## Ethics Statement

This study was approved by the Institutional Review Board and Hospital Research Ethics Committee of China Medical University (IRB permit number: CMUH-104-REC2-115).

## Author Contributions

K-AC and W-HH: designed and conceptualized study, analyzed and interpreted the data, and drafted the manuscript for intellectual content. T-YO, JS, and JW: analyzed and interpreted the data. Y-MH: designed and conceptualized study, analyzed and interpreted the data, and revised the manuscript for intellectual content. WC and C-LL: major role in the acquisition of data. All authors were involved in drafting the manuscript or revising it and approved the final version to be published.

## Conflict of Interest

The authors declare that the research was conducted in the absence of any commercial or financial relationships that could be construed as a potential conflict of interest.

## Publisher’s Note

All claims expressed in this article are solely those of the authors and do not necessarily represent those of their affiliated organizations, or those of the publisher, the editors and the reviewers. Any product that may be evaluated in this article, or claim that may be made by its manufacturer, is not guaranteed or endorsed by the publisher.
